# Remodeling of the pulmonary artery induced by metastatic gastric carcinoma: a histopathological analysis of 51 autopsy cases

**DOI:** 10.1186/1471-2407-14-14

**Published:** 2014-01-10

**Authors:** Takao Ishiwatari, Yoichiro Okubo, Naobumi Tochigi, Megumi Wakayama, Tetsuo Nemoto, Junko Kobayashi, Minoru Shinozaki, Kyoko Aki, Daisuke Sasai, Yoshiro Yamamoto, Haruo Nakayama, Kazutoshi Shibuya

**Affiliations:** 1 Department of Surgical Pathology, Toho University School of Medicine, 6-11-1, Omori-Nishi, Ota-Ku, Tokyo 143-8541, Japan; 2 Department of Neurosurgery, Toho University Ohashi Medical Center, 2-17-6, Ohashi, Meguro, Tokyo 153-8515, Japan; 3 Department of Dermatology, Peking University First Hospital, Beijing, China

**Keywords:** Remodeling, Pulmonary hypertension, Pulmonary tumor thrombotic microangiopathy

## Abstract

**Background:**

Gastric carcinoma remains the second commonest cause of cancer deaths worldwide. Presence of the carcinoma cell in the pulmonary artery is serious condition that might cause remodeling of the pulmonary artery. The present study conducted detailed histopathological analyses to elucidate how gastric carcinoma cells may affect the structure and hemodynamics of pulmonary arteries.

**Methods:**

Remodeling of the pulmonary artery was assessed based on measurements of arterial diameters and stenosis rates from the autopsies, and their correlation were also validated. We additionally calculated 95 percent confidential intervals (CIs) for the rate of stenosis in groups of pulmonary arteries of different caliber zones (under 100, 100 to 300, and over 300 micrometer). The right ventricular thickness was measured and examined whether it correlated with the rate of pulmonary arterial stenosis.

**Results:**

A total of 4612 autopsy cases were recorded at our institute, among which 168 had gastric carcinoma. Finally, 51 cases of the gastric carcinoma were employed for the study which had carcinoma cells in the lumen of the pulmonary artery. The mean right ventricular wall thickness of these cases was 3.14 mm. There were significant positive associations between the rates of pulmonary arterial stenosis and right ventricular thickness from pulmonary arteries of diameter under 100, 100 to 300, and over 300 micrometer. In these zones, 31, 31, and 33 cases had rates of pulmonary arterial stenosis that were below the lower limit of the 95 percent CI values, respectively. On the other hand, among cases with significant pulmonary stenosis, 17 of 18 cases with stenosis in the over 300 micrometer zone involved pulmonary arteries of both in the under 100 and 100 to 300 micrometer zones.

**Conclusion:**

One-third of autopsy with advanced gastric carcinoma had carcinoma cells in lumen of pulmonary artery, but implantation and proliferation may be essential to induce intimal thickening that causes an increasing of pulmonary arterial pressure, because our study revealed a significant positive association between the rate of pulmonary arterial stenosis and right ventricular thickness. In addition, diffuse type gastric carcinoma may be apt to cause the remodeling of the pulmonary artery.

## Background

Advanced diagnostic methods and therapeutic technologies have led to a steady decline in the overall mortality rate of patients with gastric carcinoma. However, the carcinoma remains the second most common cause of cancer death worldwide [1-4]. Gastric carcinoma cells has been regarded that those have high capability to metastasize to the lymph nodes and distant organs [5], which might be strongly associated with poor prognoses [6]. In particular, carcinoma cells in the pulmonary artery significantly influence tumor recurrence and death after resection [7]. These cells may also cause remodeling, especially asymmetric thickening of the intima of the pulmonary artery which can induce an increase in pressure of the right ventricle [8]. We have previously suggested that carcinoma cell in the pulmonary artery could cause remodeling of the pulmonary artery such as pulmonary tumor thrombotic microangiopathy (PTTM) on the basis of study conducted by small cohort of autopsies [9]. PTTM might be an accepted pathophysiological entity, which is characterized by tumor embolism, multiple microthrombi, and intimal myofibroblast proliferation in pulmonary arteries and arterioles. These changes may cause subsequent pulmonary hypertension [9-12]. However, the hypothesis could not be confirmed by the previous related works including our study. To determine how gastric carcinoma cells affect pulmonary arteries and that hemodynamics, we therefore conducted a detailed histopathological analysis in the present study.

## Methods

### Patients and clinical data selection

To extract autopsy cases with advanced stages of gastric carcinoma, we searched the autopsy records filed from 1981 to 2009 at the Toho University Omori Medical Center, Japan. In our medical institute, tissues from the autopsy have been fixed with 15% formalin at room temperature. Sections of formalin-fixed and paraffin-embedded tissues of lungs from the autopsy were prepared and stained with hematoxylin and eosin (H&E) and elastica van Gieson (EVG) stains. To assure accurate histopathological findings, at least three pathologists (T.I., Y.O., and K.S.) assessed each pulmonary tissue section, independently. We then obtained data regarding patient’s age and gender from medical records associated with each case. Our protocol of the present study was approved by the Ethics Committee of the Toho University School of Medicine (#23002).

### Morphometric analysis of the pulmonary artery

**Figure 1:**
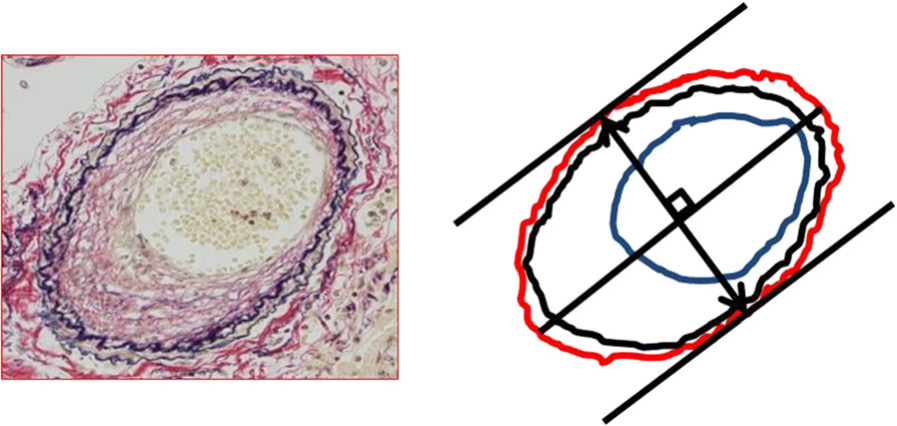
**Morphometric analysis of remodeling of the pulmonary artery.** Legend: Initially, we set the line of maximum diameter in the cross section of the pulmonary artery. Subsequently, tangent lines of external elastic lamina that parallel to the maximum diameter in the cross section of the pulmonary artery were set. And then, we set the rectangular cross line of maximum diameter in the cross section of the pulmonary artery and the distance between the tangent lines was defined as a pulmonary arterial diameter (two direction arrow). We also traced external elastic lamina (red circle line) and periphery of the residual lumen (blue circle line). The area surrounded by the former and later was defined as area within the external elastic lamina and lumen area, respectively. All measurements were conducted manually using image analyzing software (Image J 1.36b, National Institutes of Health, Bethesda, Maryland, USA).

Images of pulmonary artery with 1360×1024 pixel were taken from EVG-stained sections using a video microscope camera (DP70, Olympus, Tokyo, Japan), and each image was saved, digitally. In the present study, they were measured by the image-analyzing software (Image J 1.36b, National Institutes of Health, Bethesda, Maryland, USA) that was constituted with pulmonary arterial diameter, area within the external elastic lamina (EELA), and area of lumen (LA) based on the digitally stored images (Figure 1). To evaluate the degree of pulmonary arterial stenosis as an indicator of remodeling in the present study, we calculated the stenosis rate of the pulmonary artery according to the following formula:

$$\left(1-\left(\mathrm{LA}/\mathrm{EELA}\right)\right)\times 100=\mathrm{pulmonary}\;\mathrm{arterial}\;\mathrm{stenosis}\;\mathrm{rate}\;\left(\%\right)$$

In addition, we used the Pearson’s product–moment correlation coefficient to assess the correlation between pulmonary arterial diameter with remodeling and stenosis rate. P values < 0.05 were considered statistically significant.

### Measurement in thickness of right ventricular wall

Since it is largely accepted that right ventricular wall thickness increases with increasing in pressure of pulmonary artery (pulmonary hypertension) [13,14], we measured right ventricular thickness to know the presence and degree of pulmonary hypertension. Measurements were taken according to the following protocol.

If formalin-fixed cardiac tissue was available, we excised the posterior right ventricular wall perpendicular to the tricuspid valve ring. The excised tissues were embedded in paraffin wax. The paraffin-embedded tissues were then cut into 4 μm-thick sections and stained with H&E stain. To assess right ventricular wall thickness, we measured right ventricular thickness at three locations of the tricuspid valve 10 mm from the caspal base. Thickness of right ventricular wall was defined as the mean of these measurements. In contrast, if formalin-fixed cardiac tissue was not available, we conducted the same measurement using the cardiac tissue sections that were routinely excised at the time of diagnosis during the autopsy. To confirm that there were no differences between the former and later methods of measuring right ventricular thickness, we used a t-test to compare measurements that were taken using each method. P values < 0.05 were considered statistically significant.

### Assessment of the correlation between right ventricular wall thickness and the coincidence of basic cardiopulmonary alterations generally known as factor of increasing in pressure of pulmonary artery

It has been largely accepted that increasing in pressure of pulmonary artery occurs due to a number of causes, such as chronic obstructive pulmonary disease (including emphysema), fibrosis, cardiac valve diseases, and some kinds of drug [15-17]. We therefore assessed the tissue sections of the autopsy and checked autopsy records to confirm the coincidence of these basic cardiopulmonary alterations generally known as factor of increasing in pressure of pulmonary artery. And then, to evaluate the effect of the coincidence of these alterations, we compared the right ventricular thickness (note that it can be understood as an indicator of increasing in pressure of pulmonary artery) between patients with and without these alterations using a t-test. P values < 0.05 were considered statistically significant.

### Effect of the post mortem time interval

To evaluate the effect of the post mortem time interval in our results of investigations, we retrieved the post mortem time interval from the autopsy records. And then, the correlation between the post mortem time interval and the right ventricular thickness, stenosis rates of the pulmonary artery in the under 100 μm zone, the rates in the 100 to 300 μm zone, and the rates in the over 300 μm zone were calculated using Pearson’s product–moment correlation coefficient. P values < 0.05 were considered statistically significant.

### Assessment of the correlation between right ventricular wall thickness and remodeling of the pulmonary artery

To know the presence and degree of pulmonary hypertension, in more detail, we assessed the correlation between the rate of pulmonary arterial stenosis and right ventricular wall thickness with Pearson’s product–moment correlation coefficient, since we have observed that pulmonary arterial stenosis varied with the diameter of pulmonary arteries in patients with gastric carcinoma in our previous study [9]. Accordingly, we divided the pulmonary arteries into three zones on the basis of their caliber size to know whether caliber size affects the degree of stenosis. Following the Heath-Edwards classification [18], which has primarily been studied by Edwards et al. to evaluate the potential reversibility of pulmonary vascular disease that results from congenital cardiac septal defects. Following this system, arteries were divided into three zones based on their diameter: under 100 μm, 100 to 300 μm, or over 300 μm (these zones are referred to as the under 100 μm zone, the 100 to 300 μm zone, and the over 300 μm zone, respectively). In each zone, right ventricular wall thickness was plotted against the pulmonary arterial stenosis rates, and Pearson’s product–moment correlation coefficients were calculated. P values < 0.05 were considered statistically significant.

We were also interested in the incidence of pulmonary arterial stenosis among patients who had carcinoma cells in the lumen of the pulmonary arteries. To assess this incidence, we subdivided each of the pulmonary arterial zones into two groups: zones of pulmonary arteries with minimal or no stenosis and those with highly variable stenosis. A threshold value between the groups was employed as the lower limit of each 95% confidential interval (CI) calculated for the rate of pulmonary arterial stenosis in each arterial diameter zone. Therefore, zones of pulmonary arteries in each case falling below this threshold were considered to have minimal or no pulmonary arterial stenosis. We then calculated the average right ventricular thickness in each case. Furthermore, we compared zones using t-test to assess differences in arterial diameters (under 100 μm, 100 to 300 μm, or over 300 μm) to know an influence of distribution and/or continuity of the alterations upon an increasing in thickness of the right ventricle. P values < 0.05 were considered statistically significant.

### Assessment of the histopathological types of the gastric carcinoma

To elucidate the tumor histopathological characteristics of the 51 cases and their association with the pulmonary arteries and hemodynamics, we assessed the histopathological types of the gastric carcinoma of the autopsy in accordance with Lauren’s classification [19]. Namely, all cases were divided into intestinal type or diffuse type. And then, we investigated the ratio of intestinal and diffuse types in cases with stenosis of pulmonary arteries below and above the lower limit of the 95% CIs in each arterial diameter zone. The obtained data were analyzed statistically by the Chi-Square test and P values < 0.05 were considered statistically significant.

## Results

### Patients and clinical data selection

**Figure 2:**
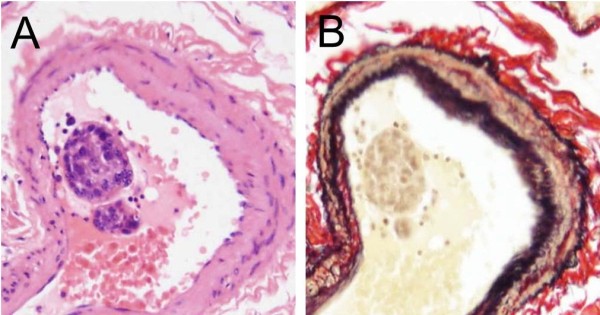
**Photomicrographs showing the lumen of the pulmonary arteries with clustered carcinoma cells.** Legend: **(A and B)** Pulmonary artery shows clustered carcinoma cells in that lumen .The cases indicating clustered carcinoma cells in the pulmonary artery were employed for the present histopathological analyses (Hematoxylin and Eosin and Elastica van Gieson stains, original magnification x 400).

Between 1981 and 2009, 4612 autopsy cases were recorded at the Toho University Omori Medical Center, of which 168 autopsy cases involving gastric carcinoma were found for the analysis in the present study. Finally, 51 cases (30.4%) from 168 autopsies with advanced gastric carcinoma were extracted of which pulmonary arteries had carcinoma cells in the lumen in clustered or sporadic (Figure 2). Ages of subjects ranged from 34 to 82 years (n = 51; mean ± standard deviation (SD): 63.5 ± 12.0). The study sample included 32 men and 19 women.

### Morphometric analysis on altered pulmonary arteries

**Figure 3:**
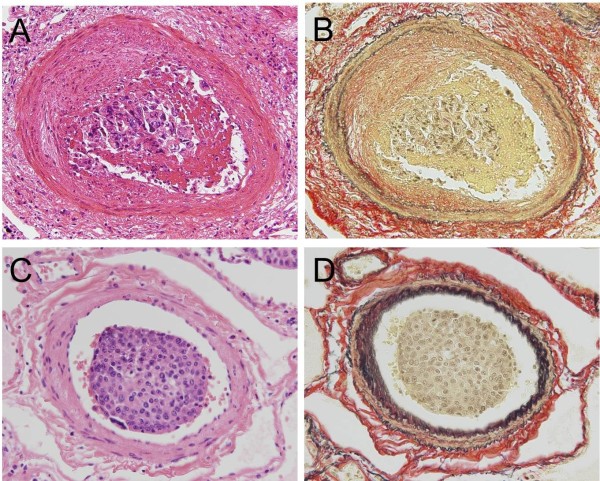
**Photomicrographs of index findings showing the pulmonary arteries with or without stenosis.** Legend: **(A and B)** The pulmonary artery of muscular type shows asymmetric intimal thickening with fibro-cellular proliferation, which is eroded and attached with clustered carcinoma cells including fibrin (Hematoxylin and Eosin (H&E) and Elastica van Gieson (EVG) stains, original magnification x 400). **(C and D)** Carcinoma cells were found in the lumen of the pulmonary artery, whereas neither fibro-cellular proliferation nor fibrin thrombus was found (H&E and EVG stains, original magnification x 400).

Even in the presence of carcinoma cells in the lumen of pulmonary artery in the investigated cases, the degree of remodeling of them varied from case to case. Namely, some pulmonary arteries showed asymmetric intimal thickening with fibro-cellular proliferation, where is eroded and covered with fibrin including carcinoma cells (Figure 3A and B). Conversely, some pulmonary arteries showed neither intimal thickening nor fibrin thrombus (Figure 3C and D). There was no significant correlation between pulmonary arterial diameters and stenosis rate in large body of the subjects (38 cases), and 6 and 7 cases from remainders indicated significant positive and negative correlations, respectively (Table 1).

**Table 1:** Pearson product–moment correlation coefficients (r) between the pulmonary arterial diameter and the rate of stenosis

**Case**	**r**	**Case**	**r**
1	NS	27	NS
2	-0.20	28	NS
3	NS	29	-0.16
4	0.26	30	0.22
5	NS	31	NS
6	NS	32	NS
7	NS	33	NS
8	NS	34	0.18
9	NS	35	NS
10	NS	36	NS
11	NS	37	NS
12	NS	38	-0.20
13	NS	39	NS
14	NS	40	NS
15	NS	41	0.31
16	NS	42	NS
17	NS	43	NS
18	NS	44	0.18
19	NS	45	-0.43
20	NS	46	-0.56
21	NS	47	-0.42
22	NS	48	NS
23	NS	49	NS
24	NS	50	-0.30
25	NS	51	NS
26	0.34		

Legend: In each of the 51 autopsy cases, the Pearson product–moment correlation coefficient between pulmonary arterial diameters and stenosis rate was conducted. Results indicated significant positive correlations for six cases, significant negative correlations for seven cases, and no significant correlation for the remaining 38 cases.

### Measurement in thickness of right ventricular wall

Among our cases, 24 autopsies included preserved formalin-fixed cardiac tissues, which enabled us to newly excise the cardiac tissue to measure the right ventricular wall thickness. Formalin-fixed cardiac tissues were not available for the remaining 27 cases and we therefore relied on cardiac tissue sections, which were routinely excised prior to the present study. The means ± SD of right ventricular wall thickness in the former and latter groups was 3.33 ± 0.94 mm and 3.08 ± 0.87 mm, respectively. There was no significant difference in thickness between these groups. The mean right ventricular wall thickness of these 51 cases was 3.14 ± 0.90 mm.

### Assessment of the correlation between right ventricular wall thickness and the coincidence of basic cardiopulmonary alterations generally known as factor of increasing in pressure of pulmonary artery

Among our cases, nine patients with emphysema and two patients with interstitial pneumonia were found. On the other hand, neither patient with cardiac valve diseases nor patient treated with drugs that cause increasing in pressure of pulmonary artery was found in the present study. These data were summarized in Table 2. The means ± SD of right ventricular wall thickness in patients with and without emphysema was 3.54 ± 0.88 mm and 3.05 ± 0.88 mm, respectively. There was no significant difference in thickness between these groups (t-test, p = 0.133). Similarly, the means ± SD of right ventricular wall thickness in patients with and without interstitial pneumonia was 3.15 ± 1.05 mm and 3.13 ± 0.90 mm, respectively. There was also no significant difference in thickness between these groups (t-test, p = 0.982)

**Table 2:** The coincidence of basic cardiopulmonary alterations generally known as factor of increasing in pressure of pulmonary artery

**Case**	**COPD**	**IP**	**CVD**	**Drugs**	**Case**	**COPD**	**IP**	**CVD**	**Drugs**
1	-	-	-	-	27	-	-	-	-
2	+	-	-	-	28	-	-	-	-
3	-	-	-	-	29	-	-	-	-
4	-	-	-	-	30	-	-	-	-
5	-	-	-	-	31	-	-	-	-
6	-	-	-	-	32	-	-	-	-
7	+	-	-	-	33	-	-	-	-
8	+	-	-	-	34	-	-	-	-
9	-	-	-	-	35	-	-	-	-
10	-	-	-	-	36	-	-	-	-
11	-	-	-	-	37	-	-	-	-
12	-	-	-	-	38	-	-	-	-
13	-	-	-	-	39	+	-	-	-
14	-	-	-	-	40	-	-	-	-
15	-	-	-	-	41	+	-	-	-
16	+	-	-	-	42	-	-	-	-
17	-	-	-	-	43	-	-	-	-
18	-	-	-	-	44	-	-	-	-
19	-	-	-	-	45	-	-	-	-
20	+	+	-	-	46	-	-	-	-
21	-	-	-	-	47	+	-	-	-
22	-	-	-	-	48	-	+	-	-
23	-	-	-	-	49	-	-	-	-
24	-	-	-	-	50	-	-	-	-
25	+	-	-	-	51	-	-	-	-
26	-	-	-	-					

COPD: Chronic obstructive pulmonary disease, IP: Interstitial pneumonia, CVD: Cardiac valve diseases, Drugs: aminorex, cocaine, dexfenfluramine, fenfluramine, phenylpropanolamine, and selective serotonin reuptake inhibitors.

Legend: Nine patients with emphysema and two patients with interstitial pneumonia were found. On the other hand, neither patient with cardiac valve diseases nor patient treated with drugs that cause increasing in pressure of pulmonary artery.

### Effect of the post mortem time interval

The mean ± SD of the post mortem time interval in the autopsy was 384.78 ± 346.23 minute. There were no significant differences between the post mortem time interval and the right ventricular thickness, stenosis rates of the pulmonary artery in the under 100 μm zone, the rates in the 100 to 300 μm zone, and the rates in the over 300 μm zone (Pearson’s product–moment correlation coefficient, P = 0.428, 0.064, 0.107, and 0.104, respectively).

### Assessment of the correlation between right ventricular wall thickness and remodeling of the pulmonary artery

**Figure 4:**
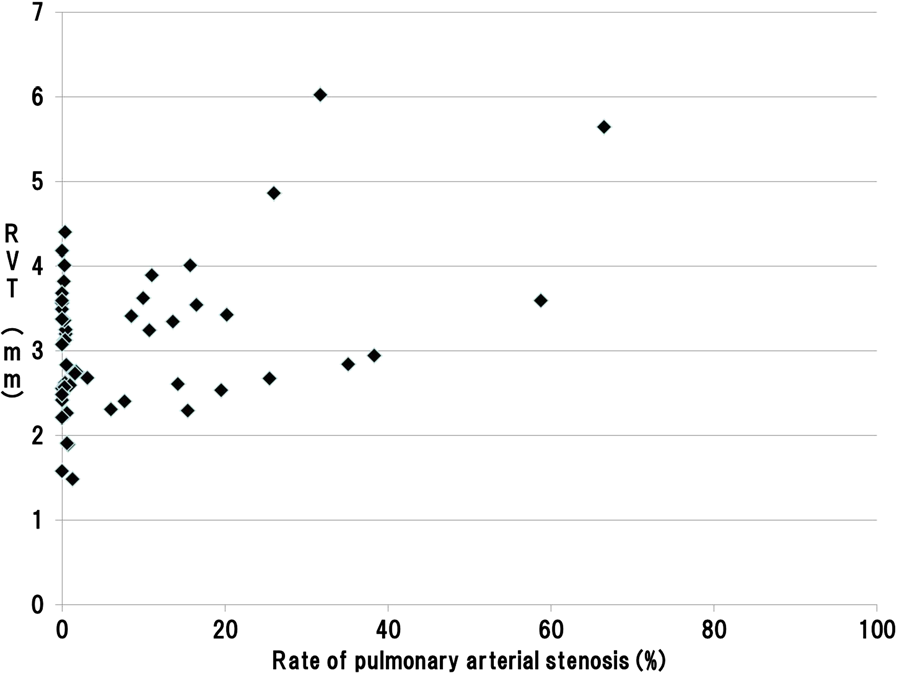
**Scatter plot of the pulmonary arterial stenosis rate and RVT in the under 100 μm zone.** Legend: A significant positive association was found between pulmonary arterial stenosis rates and right ventricular thickness in the under 100 μm zone; the correlation coefficient was 0.442. (Pearson product–moment correlation coefficient, p < 0.001) (RVT: Right Ventricular wall Thickness).

**Figure 5:**
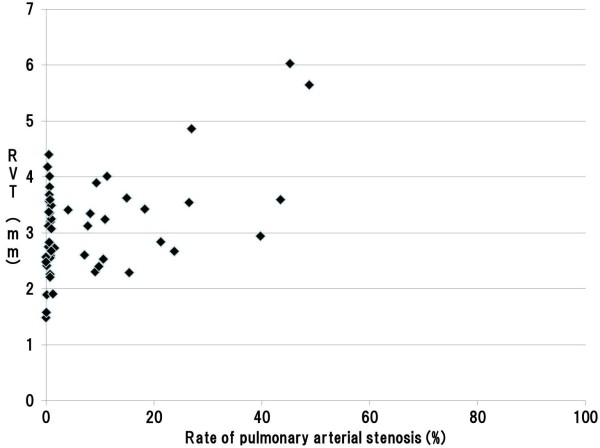
**Scatter plot of pulmonary arterial stenosis rate and RVT in the 100 to 300 μm zone.** Legend: A significant positive association was found between pulmonary arterial stenosis rates and right ventricular thickness in the 100 to 300 μm zone; the correlation coefficient was 0.515. (Pearson product–moment correlation coefficient, p < 0.001) (RVT: Right Ventricular wall Thickness).

**Figure 6:**
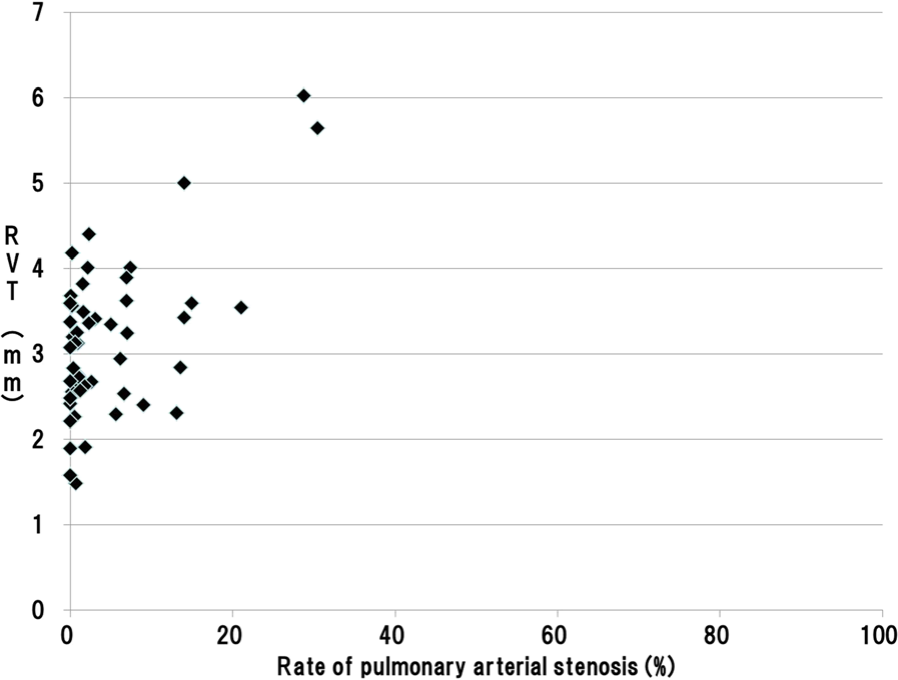
**Scatter plot of the pulmonary arterial stenosis rate and RVT in the over 300 μm zone.** Legend: A significant positive association was found between pulmonary arterial stenosis rates and right ventricular thickness in the over 300 μm zone; the correlation coefficient was 0.592. (Pearson product–moment correlation coefficient, p < 0.001) (RVT: Right Ventricular wall Thickness).

The 95% CIs for stenosis rates of the pulmonary artery were 4.97–13.36% in the under 100 μm zone, 4.87–12.14% in the 100 to 300 μm zone, and 2.71–6.71% in the over 300 μm zone, respectively. There was 31, 31, and 33 cases exhibited minimal or no pulmonary arterial stenosis in each of the caliber zones, respectively (Tables 3, 4, and 5). On the other hand, among cases with significant pulmonary stenosis, 17 of 18 cases with stenosis in the over 300 μm zone involved pulmonary arteries of both in the under 100 μm and 100 to 300 μm zones. We therefore compared the right ventricular wall thicknesses in cases with stenosis of pulmonary arteries below and above the lower limit of the 95% CIs. For all zones of caliber, we found that right ventricular wall thickness was significantly greater among cases above the lower limit of the 95% CI index (Tables 3, 4, and 5). In addition, our assessment on the correlation between pulmonary arterial stenosis rates and right ventricular thickness using the Pearson’s product–moment correlation coefficient indicated a significant positive association in each zone; in the under 100 μm, 100 to 300 μm, and over 300 μm zones, with the correlation coefficients of 0.442, 0.515, 0.592, respectively (Figures 4, 5, and 6).

**Table 3:** Differences of the right ventricular wall thickness in the under 100 μm zone

	**Below the lower limit of the 95% CI**	**Above the lower limit of the 95% CI**
Case number	31	20
The mean of RV (mm)	2.97	3.96

Legend: The 95% confidential interval (CI) for stenosis rates of the pulmonary artery under 100 μm zone was 4.97 to 13.36%. In this zone, 31 cases were less than the lower limit of the 95% CI. In addition, the right ventricular wall thickness was significantly greater among cases above the lower limit of the 95% CI (p = 0.038, t test).

RVT: Right ventricular wall thickness.

**Table 4:** Differences of the right ventricular wall thickness in the 100 to 300 μm zone

	**Below the lower limit of the 95% CI**	**Above the lower limit of the 95% CI**
Case number	31	22
The mean of RV (mm)	2.94	3.44

Legend: The 95% confidential interval (CI) for stenosis rates of the pulmonary artery from 100 to 300 μm zone was 4.87 to 12.14%. In this zone, 31 cases were less than the lower limit of the 95% CI. In addition, the right ventricular wall thickness was significantly greater among cases above the lower limit of the 95% CI (p = 0.048, t test).

RV: Right ventricular wall thickness.

**Table 5:** Differences of the right ventricular wall thickness in the over 300 μm zone

	**Below the lower limit of the 95% CI**	**Above the lower limit of the 95% CI**
Case number	33	18
The mean of RV (mm)	2.91	3.55

Legend: The 95% confidential interval (CI) for stenosis rates of the pulmonary artery over 300 μm zone was 2.71 to 6.71%. In this zone, 33 cases were less than the lower limit of the 95% CI. In addition, the right ventricular wall thickness was significantly greater among cases above the lower limit of the 95% CI (p = 0.013, t test).

RV: Right ventricular wall thickness.

### Assessment of the histopathological types of the gastric carcinoma

In the groups of pulmonary artery under 100 μm and 100 to 300 μm zones, cases with stenosis of pulmonary arteries below the lower limit of the 95% CIs had 21 cases of intestinal type gastric carcinoma and 10 cases of diffuse type one. On the other hand, cases with stenosis of pulmonary arteries above the lower limit of the 95% CIs had 4 cases of intestinal type gastric carcinoma and 16 cases of diffuse type one.

In the group of pulmonary artery over 300 μm zones, cases with stenosis of pulmonary arteries below the lower limit of the 95% CIs had 21 cases of intestinal type gastric carcinoma and 12 cases of diffuse type one. On the other hand, cases with stenosis of pulmonary arteries above the lower limit of the 95% CIs had 4 cases of intestinal type gastric carcinoma and 14 cases of diffuse type one. In addition, our statistical analysis revealed that the ratio of diffuse type gastric carcinoma was significantly higher than the ratio of intestinal type one in cases with stenosis of pulmonary arteries above the lower limit of the 95% CIs in each arterial diameter zone (Chi-Square test, in the under 100 μm, 100 to 300 μm, and over 300 μm zones, P < 0.001, P < 0.001, P = 0.005, respectively). These data were summarized in Table 6.

**Table 6:** Histopathological types of gastric carcinoma in cases with below and above the lower limit of the 95% CI in each group of pulmonary arterial zone

	**Pulmonary artery under 100 μm zone**		**Pulmonary artery 100 to 300 μm zone**		**Pulmonary artery over 300 μm zone**	
Type*	**Intestinal**	**Diffuse**	**Intestinal**	**Diffuse**	**Intestinal**	**Diffuse**
Below the lower limit of the 95% CI	21	10	21	10	21	12
Above the lower limit of the 95% CI	4	16	4	16	4	14
P value (Chi-Square test)	P < 0.001		P < 0.001		P = 0.005	

Legend: In the groups of pulmonary artery under 100 μm and 100 to 300 μm zones, cases with stenosis of pulmonary arteries below the lower limit of the 95% CIs had 21 cases of intestinal type gastric carcinoma and 10 cases of diffuse type one. On the other hand, cases with stenosis of pulmonary arteries above the lower limit of the 95% CIs had 4 cases of intestinal type gastric carcinoma and 16 cases of diffuse type one.

In the group of pulmonary artery over 300 μm zones, cases with stenosis of pulmonary arteries below the lower limit of the 95% CIs had 21 cases of intestinal type gastric carcinoma and 12 cases of diffuse type one. On the other hand, cases with stenosis of pulmonary arteries above the lower limit of the 95% CIs had 4 cases of intestinal type gastric carcinoma and 14 cases of diffuse type one. In addition, our statistical analysis revealed that the ratio of diffuse type gastric carcinoma was significantly higher than the ratio of intestinal type one in cases with stenosis of pulmonary arteries above the lower limit of the 95% CIs in each arterial diameter zone (Chi-Square test, in the under 100 μm, 100 to 300 μm, and over 300 μm zones, P < 0.001, P < 0.001, P = 0.005, respectively).

*: “Type” indicated in the table means each histological type of gastric carcinoma proposed by Lauren [19].

## Discussion

Distant metastasis has been significantly associated with poorer survival among patients with gastric carcinoma [20]. In particular, carcinoma cells in the pulmonary artery can cause fatal pulmonary complications [7,9,21]. Therefore, we have investigated the part of this fatal complication, which could be regarded as PTTM. Our previous study could suggest that the presence of carcinoma cells itself may not lead to pulmonary hypertension, certainly, and most of cases with sudden death might be caused by acute thromboembolic occlusion of the pulmonary arteries triggered by the circulation of carcinoma cells [9]. In addition, an increasing of pulmonary arterial pressure might be induced by completion of continuous intimal thickening by implantation of carcinoma cells with increasing of matrix in the intima. However, it had not enabled to provide defined conclusion announcing the hypothetical considerations due to that small number of subjects. Therefore, we conducted the present histopathological analysis using more than 50 subjects exhibiting carcinoma cells in the lumen of the pulmonary artery.

Results indicate that gastric carcinoma cells in the pulmonary artery may cause remodeling of the pulmonary artery, but the presence or absence of luminal stenosis varies from case to case. In addition, since 17 of 18 cases with stenosis in the over 300 μm zone also had remodeling of pulmonary arteries of both in the under 100 μm and 100 to 300 μm caliber zones, it can be understood that remodeling of pulmonary artery induced by implantation of gastric carcinoma cells that causes an increasing of pulmonary arterial pressure may simultaneously occur in wide range of arterial caliber. Therefore, it emerged from our study that manner, especially distribution and extension of intimal thickening induced by implantation of metastatic carcinoma cells differs from that observed in patients with idiopathic pulmonary arterial hypertension, a condition that initially cause pulmonary arterial stenosis in small muscular arteries and arterioles in the lung [14,22,23].

On the other hand, it should be another interest to consider the correlation between remodeling of the pulmonary artery and pulmonary hypertension. Whereas we also investigated the correlation between different caliber zones with luminal stenosis in the pulmonary artery and the right ventricular wall thickness to know an influence of difference in caliber involved upon the degree of pulmonary hypertension, our result revealed that all cases with remodeling in pulmonary arteries in any caliber zones caused pulmonary hypertension, which was confirmed by an increasing in thickness of right ventricular wall.

We here wish to refer to the background of the autopsy. In the present study, there was no significant correlation between the post mortem time interval and right ventricular thickness, as well as the interval and the stenosis rates of pulmonary artery in each pulmonary arterial zone. These facts indicated that post mortem time interval of the autopsy has no effect on the results of our investigations. Furthermore, some alterations that known as factor of increasing in pressure of pulmonary artery (nine patients with emphysema and two patients with interstitial pneumonia) were found, but there were no significant differences in right ventricular thickness in patients with or without these alterations. This fact indicated that the presence of emphysema and interstitial pneumonia had no effect on the back-ground lung structures in the present study.

Further discussion is warranted regarding the histopathological types of gastric carcinoma in the present study. It has been reported that diffuse type (poorly differentiated) gastric carcinoma tends to cause intimal thickening of the pulmonary artery [9,11,24]. We therefore investigated the histopathological types of the gastric carcinoma of 51 cases in accordance with Lauren’s classification [19]. Our investigations revealed that the ratio of diffuse type gastric carcinoma was significantly higher than the ratio of intestinal type one in cases with stenosis of pulmonary arteries above the lower limit of the 95% CIs in each group of pulmonary arterial diameter zone. This fact indicated that diffuse type gastric carcinoma may be apt to cause the remodeling of the pulmonary artery and subsequent increasing in pressure of pulmonary artery.

## Conclusion

We found carcinoma cells in the lumen of pulmonary artery in one-third (30.4%) of autopsy with advanced gastric carcinoma, but implantation and proliferation may be essential to induce intimal thickening that causes an increasing of pulmonary arterial pressure, because our study revealed a significant positive association between the rate of pulmonary arterial stenosis and right ventricular thickness. In addition, diffuse type gastric carcinoma may be apt to cause the remodeling of the pulmonary artery and subsequent increasing in pressure of pulmonary artery.

## Abbreviations

PTTM: Pulmonary tumor thrombotic microangiopathy; HE: Hematoxylin and eosin; EVG: Elastica van Gieson; EELA: Area within the external elastic lamina; LA: Lumen area; CI: Confidential interval; SD: Standard deviation.

## Competing interests

Dr. Shibuya reports receiving research grants from Janssen Pharmaceutical K.K., Dainippon Sumitomo Pharma Co., Astellas Pharma Inc., Taiho Pharmaceutical Co., and POLA-Pharma Inc. Other authors declare that they have no competing interests.

## Authors’ contributions

TI integrated the data and completed the manuscript as a major contributor; YO conceptualized the report, integrated the data, carried out statistical evaluation, revised the manuscript, and gave final approval to the manuscript as a corresponding author; NT evaluated the histopathological types of primary lesion (stomach) and integrated data, MW evaluated the remodeling of the pulmonary artery and partially evaluated the primary lesion, TN carried out the histopathological examinations in primary and pulmonary lesions and revised histopathological description; JK integrated data partially from the medical records of the autopsy, SM carried out a part of histopathological examinations and extracted raw data from autopsy records, AK carried out extraction of raw data from autopsy records and carried out a part of histopathological examinations, DS integrated a part of data obtained from autopsy records, YY integrated a part of data obtained from autopsy records and partially carried out histopathological examination of the primary lesions, HN carried out a part of histopathological examinations and statistical evaluation, KS integrated the data, revised manuscript, carried out histopathological examinations as a last author. Furthermore, all authors contributed towards the conceptualization, writing, reading, and approval of the final manuscript.

## Pre-publication history

The pre-publication history for this paper can be accessed here:

http://www.biomedcentral.com/1471-2407/14/14/prepub
